# Clustering of non-invasive biomarkers to distinguish phenotype-related response differences in women with PCOS after the 16-week Nordic interval training intervention: A prospective cohort study

**DOI:** 10.1016/j.jesf.2026.200499

**Published:** 2026-07-17

**Authors:** Zahra Arab Taheri Zadeh, Valiollah Dabidi Roshan, Marzieh Mirzaei

**Affiliations:** a Department of Exercise Physiology, Faculty of Sports Sciences, University of Mazandaran, Babolsar, Iran; b Athletic Performance and Health Research Centre, Faculty of Sports Sciences, University of Mazandaran, Babolsar, Iran; c Tehran Medical Branch, Islamic Azad University of Tehran, Shariati St., Tehran, 1916893813, Iran

**Keywords:** Explainable artificial intelligence, PCOS, Healthy lifestyle, Physical activity, Anti-mullerian hormone

## Abstract

**Background:**

Polycystic ovary syndrome (PCOS) is associated with metabolic and reproductive abnormalities. Response variability due to exercise may reflect underlying biological heterogeneity in PCOS phenotypes. We explored changes in non-invasive PCOS biomarkers following a 16-week Interval Nordic Walking (INW) to identify phenotype-related response differences.

**Methods:**

A total of 7500 women's data were obtained from the Yazd Reproductive Sciences and Amir al Mómenin Hospital (2019-2024). PCOS was diagnosed according to the Rotterdam criteria. XGBoost feature selection was implemented to choose 17 key predictors. Multiple ML classifiers were evaluated, and model interpretability was achieved using SHAP values. 80 women with different PCOS phenotypes completed a 16-week INW intervention. PCA was applied to reduce the features to 75% variance. Then clustering algorithms (K-means, GMM, Hierarchical, Spectral, DBSCAN) were evaluated using Silhouette scores, Calinski-Harabasz indices, and bootstrap resampling (Jaccard index).

**Results:**

XGBoost and SHAP analysis confirmed AMH, FSH, and Anti-TPO as the most influential predictors of PCOS, while metabolic indices like HbA1c, BMI, and lipid profiles exhibited moderate but clinically relevant effects. The spectral model provided better separation among evaluated algorithms; however, overall cluster separation remained modest. It suggested two exploratory data-driven response patterns, termed high-response (more frequently observed phenotypes one and two), which demonstrated greater observed reductions in testosterone and DHEA-s along with improvements in HDL and total cholesterol following the intervention. Low-response (more frequently observed phenotypes three and four) showed relatively minimal response to INW. Based on mean Jaccard index = 0.45, cluster stability was moderate.

**Conclusions:**

Our study suggests the heterogeneous phenotypic response pattern of PCOS following a 16-week INW intervention and supports further investigation of nonpharmacological strategies for PCOS.

## 1 Introduction

Polycystic ovarian syndrome (PCOS) is a very common endocrine disorder that affects 4–20% of women of reproductive age worldwide.^1^ It is a biologically heterogeneous condition with distinct phenotypic expressions that vary in metabolic disorders and androgen excess.^2^ Emerging evidence indicates that metabolic disturbances are more prevalent in hyperandrogenic phenotypes compared to normoandrogenic ones,^2^ suggesting that PCOS biological heterogeneity is important for therapeutic implications, while most studies treat PCOS as a homogeneous condition without considering each phenotype's response to intervention.

In recent years, conventional statistical approaches have improved our understanding of PCOS pathophysiology; however, they often fail to capture complex nonlinear interactions among reproductive hormones, metabolic biomarkers, and anthropometric indices.^3^ Machine learning (ML) techniques, especially explainable AI and unsupervised clustering, are considered a robust alternative for identifying high-dimensional biomarkers and latent patterns without strict parametric constraints.^4^^,^^5^ In this regard, unsupervised clustering approaches have also been used to identify cardiometabolic and reproductive subtypes of PCOS in cross-sectional cohorts.^5^^,^^6^ Nevertheless, these studies have largely focused on baseline phenotyping and have not examined exercise-induced longitudinal response patterns. However, no previous study has investigated integrated unsupervised ML-based clustering of exercise response with an INW intervention to measure the extent of PCOS phenotypes' response.

Regular exercise is a cornerstone of PCOS management, known to improve insulin sensitivity, lipid metabolism, reproductive hormone balance, and psychological well-being.^7^ However, despite evidence of the benefits of exercise, traditional studies have poorly considered the PCOS phenotypes and often investigated the effect of interventions on PCOS regardless of phenotypes.^8^ Emerging evidence indicates the aforesaid metabolic disturbances are more prevalent in hyperandrogenic phenotypes compared to normoandrogenic ones,^2^ suggesting that different phenotypes may exhibit distinct responses to exercise interventions. Hence, understanding phenotype-specific changes to exercise is essential for developing precision lifestyle medicine strategies in PCOS management. Despite growing evidence supporting regular exercise for PCOS, the impact of INW on the multidimensional phenotypic structure of the syndrome has not yet been investigated.

To our knowledge, no previous study has investigated integrated unsupervised ML-based clustering of exercise response with an INW intervention to measure the extent of PCOS phenotypes' response. Thus, we employed unsupervised clustering as an exploratory tool to identify response patterns. Furthermore, explainable AI techniques (XGBoost and SHAP) were used to identify biomarker signatures for the exploratory response patterns. Additionally, as mentioned above, the moderate Jaccard stability (mean = 0.45) suggests that any boundary between phenotypes may be fluid rather than rigid patterns. We hypothesized that differences in exercise responses may partly reflect underlying biological heterogeneity among PCOS phenotypes rather than merely statistical noise or limitations of the model. However, small size and measurement variability may cause different observations, based on evidence, distinct endocrine and metabolic profiles characterize phenotypes of PCOS.^9^ Therefore, we sought to explore changes in non-invasive biomarkers following a 16-week INW intervention and distinguish phenotype-related response differences in Iranian women with PCOS.

## 2 Methods

### 2.1 Study design and ethical considerations

The study was designed as a mixed-methods approach, including 3 phases:

- Phase 1: **A retrospective observational phase** for biomarker selection and validation of their predictive value in a controlled intervention
- Phase 2: **A prospective, single-arm intervention phase** to explore biomarker changes observed following participation in the INW program.
- Phase 3: **An exploratory, unsupervised machine learning analysis** to identify the pattern of exercise response in PCOS phenotypes

**Fig. 1:**
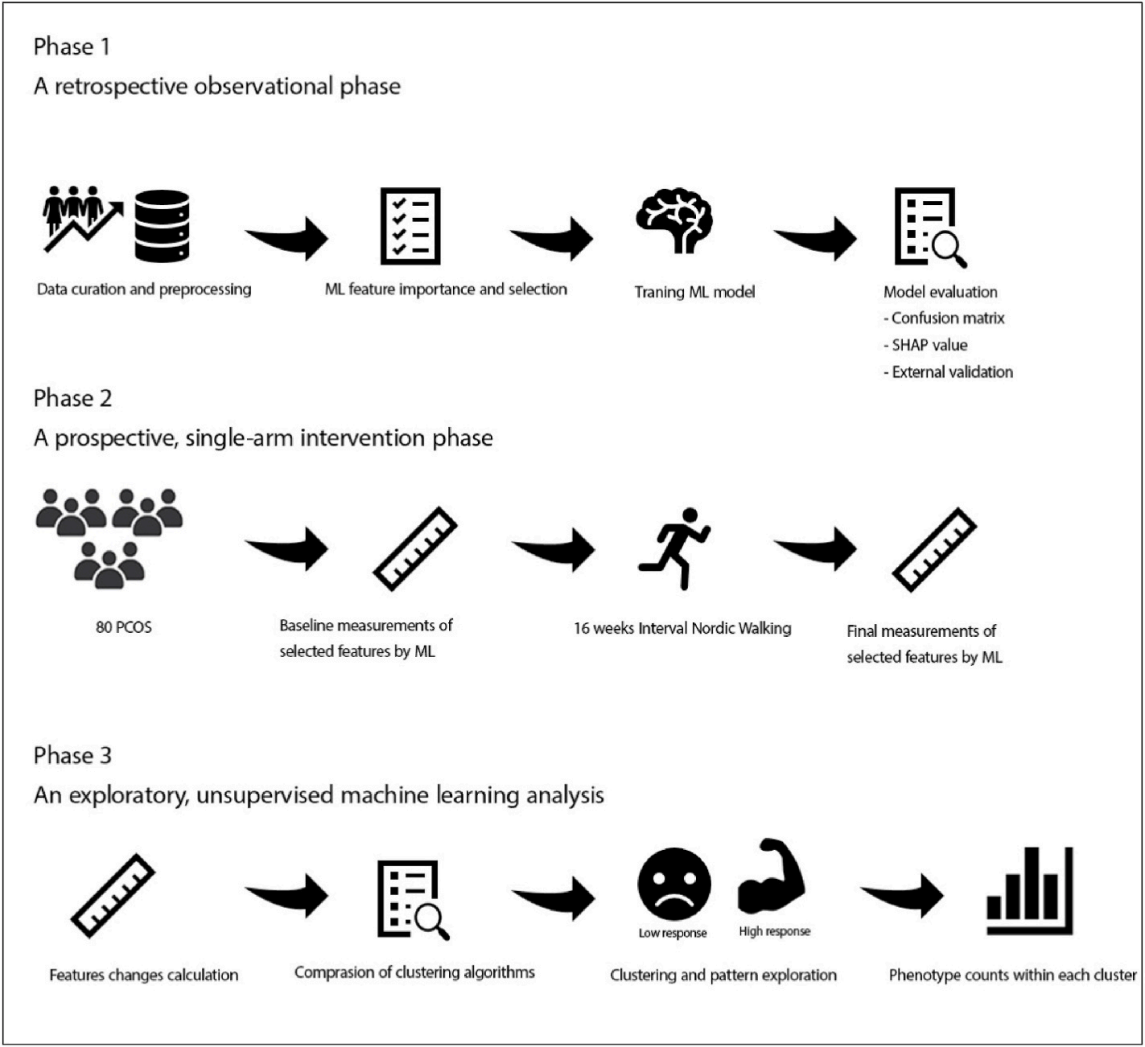
Study design in the current research.

The study protocol was approved by the Ethics Committee and was conducted in accordance with the Helsinki Declaration. In this study, clinical diagnosis of PCOS is based on the Rotterdam criteria, which comprises oligo/anovulation, polycystic ovaries, and hyperandrogenism. Infertility is defined as the failure to conceive after 12 months or more of regular sexual intercourse.^10^ The study design is presented in Fig. 1.

## 3 Phase 1: A retrospective observational phase

### 3.1 Data curation for classifier models

Initially, a total of 7500 samples of Iranian women with and without PCOS (aged between 20 and 49 years), each containing 120 features, were received from the Yazd Reproductive Sciences Institute between 2019 and 2024. All personal identifiers were removed before analysis, and the dataset was fully anonymized; therefore, patient consent was not required according to international ethical guidelines for secondary data use.^11^

### 3.2 Data preprocessing

#### 3.2.1 Removal of irrelevant or redundant variables

Non-informative variables, including marital status, patient IDs, spouse's age, and long-term medical history, were excluded to avoid noise in the downstream modeling pipeline.^12^ Data samples and feature distributions are shown in the supplementary file in Table S.I.

#### 3.2.2 Outlier assessment

Visual and statistical inspection using Python (boxplots, z-score analysis) revealed no extreme outliers; thus, all remaining samples were retained.

#### 3.2.3 Handling missing data

To manage missing data, statistical approaches were used based on the amount of data missing and the feature's importance. Traditional statistical methods, such as mean, mode, and median, were used when the proportion of missing data is 5% to 10%,^13^ and sophisticated approaches, such as hot-deck and K-Nearest Neighbors (KNN) Imputation, were used when the proportion of missing data is 20% to 30%.^14^ In our dataset, features with more than 30% missing values, such as abortion, progesterone, and βHCG, were removed.^15^ For categorical features, missing values were replaced with the mode, and for the numerical features, missing values were replaced with the mean.^14^

Finally, the dataset comprises 658 infertile and fertile instances (Age: 34.7 ± 6.3; BMI: 27.7 ± 4.67) and 27 attributes, of which 320 instances were in the positive class (PCOS women), and 338 were in the negative class (non-PCOS women). The difference between the two classes is negligible; therefore, class balancing is not required. Collected data include Age, Weight, Height, BMI, Abnormal ovaries, Ovarian cysts, Dysmenorrhea, Amenorrhea, Infertility, Irregular menstruation, signs of Hyperandrogenism, Acne, Hirsutism, AMH, FSH, LH, Prolactin, TSH, Anti-TPO, HbA1c, FBS, TG, Cholesterol, LDL, HDL, and Vitamin D3.

#### 3.2.4 Data encoding

Machine learning algorithms require numerical representations of categorical features. Since the dataset consists of some categorical features like infertile (yes/no) or having a regulated menstrual cycle (yes/no), we encoded all categorical features with the LabelEncoder of the Scikit-learn library. The categorical features with Y and N have been encoded as 1 and 0, respectively, and the ‘PCOS(Y/N)’ variable has been set as the target variable.

#### 3.2.5 Standardization

To ensure equal contribution of all numerical features and to improve convergence of gradient-based algorithms, standardization was performed using Scikit-learn's StandardScaler, which transforms features' means into 0 and standard deviations into 1. The StandardScaler's mathematical representation is given in Equation (1), where X_i_ is the original feature, *X*_*new*_ is the standardized feature, *X*_*mean*_ is the mean of the feature, and *X*_*sd*_ is the standard deviation of the feature.

(1) $$X_{new}=\left(X_{i}-X_{mean}\right)/X_{sd}$$

### 3.3 Feature selection

Feature selection removes irrelevant features to choose an optimal subset. Feature selection techniques are generally categorized into filter, wrapper, embedded, and hybrid, which is the combination of these techniques.^13^ XGBoost is a tree-based algorithm composed of several base learners. This algorithm was chosen because it ranks the features based on real-world prediction error reduction.^16^

Feature importance was initially measured using the weight metric (frequency of splits across trees), enabling the extraction of the most diagnostically significant predictors for PCOS.

### 3.4 Machine learning models

Several models, like K-Nearest Neighbors (KNN), Logistic Regression (LR), Support Vector Classifier (SVC), XGBoost, Decision Tree (DT), AdaBoost, and Random Forest (RF) classifiers, were employed.

### 3.5 Data splitting

The PCOS dataset was split into two sets. 80% was used for training and optimizing the model, and 20% was used to evaluate and test the model.

### 3.6 Optimization (grid search CV optimization technique)

Grid search is a tool from scikit-learn that evaluates all combinations of possible parameters through cross-validation to choose the optimal set.^17^

### 3.7 Evaluating models

Trained models were evaluated on test data to assess their diagnosis performance for PCOS. Model efficiency was evaluated using the confusion matrix, and performance metrics including *accuracy, precision, recall, specificity*, and *F1-score* were computed according to Equations (2)–(6):

(2) Accuracy=(TP+TN)/(TP+FP+TN+FN)

(3) Precision=TP/(TP+FP)

(4) Recall=TP/(TP+FN)

(5) F1−score=2×((precision×recall)/(precision+recall))

(6) Specificity=TN/(FP+TN)

Here, TP indicates true positive, TN indicates true negative, FP indicates false positive, and FN indicates false negative. We also used the receiver operating characteristic (ROC) curve to evaluate the binary classification model's performance in a graphical form. The area under the curve (AUC) specifies the degree of separability, and a higher AUC value demonstrates better classifier performance.^13^

### 3.8 External validation

External validation provides an unbiased estimation of model capability. To evaluate the proposed model, data from 200 individuals (100 PCOS and 100 non-PCOS; Age: 35.49 ± 5.06; BMI: 28.4 ± 0.28) sourced from the Amir al-Mómenin Hospital were collected from 2024 to 2025. External data were completely independent and were not involved in the model training and internal testing.

Model performance was assessed on this dataset using the same evaluation metrics. The results are presented in Table 1.

**Table 1:** Metric evaluation of models based on the top 17 selected features by XGBoost.

Models	Specificity %	Accuracy %	Precision %	Recall %	F1-Score %	AUC %
XGBoost	90	94	92	97	95	**98**
KNN	92	82	92	74	82	**92**
LR	90	90	92	90	91	**93**
DT	90	90	92	90	91	**87**
AdaBoost	86	92	90	97	93	**97**
RF	88	92	91	95	93	**98**
SVC	92	92	93	93	93	**95**
XGBoost (External validation)	99	92	92	97	95	**91**

KNN, K-Nearest Neighbors; LR, Logistic Regression; DT, Decision Tree; RF, Random Forest; SVC, Support Vector Classifier.

## 4 Phase 2: A prospective, single-arm intervention phase

### 4.1 Data curation and exercise intervention

80 Iranian obese women with PCOS (BMI: 31 ± 1.9 kg/m^2^; aged 23-33 years) were recruited based on the Rotterdam criteria phenotype, including:

- Phenotype one (oligo/anovulation, hyperandrogenism, and polycystic ovaries);
- Phenotype two (oligo/anovulation and hyperandrogenism);
- Phenotype three (hyperandrogenism and polycystic ovaries);
- Phenotype four (anovulation and polycystic ovaries).^18^

The exclusion criteria consist of: performance of regular physical exercise at least three times per week, smoking, pregnancy, Cushing's syndrome, diabetes, thyroid diseases, congenital adrenal hyperplasia, and hyperprolactinemia.^19^ Participants were stratified by their phenotypes into equal groups, and 17 features selected by XGBoost were evaluated at baseline and after 16 weeks of interval Nordic walking (INW). The INW protocol was adapted from previous studies with some modifications.^20^ In summary, based on a pilot study, 3000 steps were considered basic steps. The Pedometer, model Walking style II, manufactured by the OMRON company, was utilized. Participants walked three days a week for four months. The principle of progressive overload was considered, so that subjects in the 40th training session were able to walk 7500 steps in 30 min. Furthermore, the heart rate was maintained in the range of 60-75% HRmax with a heart rate monitor (Polar AXN500, Finland).

### 4.2 Intervention adherence and compliance

Initially, 80 volunteers were recruited. During the 16-week INW, 28 patients were withdrawn (phenotype: one = 6, phenotype two = 6, phenotype three = 9, phenotype four = 7) due to illness, irregular attendance, dissatisfaction with participation, or failure to meet the predefined adherence criterion. Adherence percentage was estimated as the percentage of attended exercise sessions relative to the total number of prescribed sessions:

(7) Attendance(%)=(Numberofattendedsessions/totalprescribedsessions)×100

Participants who attended less than 87% of the 48 sessions were excluded. Furthermore, withdrawn participants were replaced by newly recruited individuals from the same PCOS phenotype group and the same baseline assessments to maintain equal sample sizes across PCOS phenotypes, and each subsequently completed a 16-week INW (total 80 patients; each phenotype group, n = 20). Finally, 80 participants completed the 16-week INW intervention and all adherence requirements.

Direct supervision and heart rate monitoring were used to maintain intensity in the range of 60-75% HRmax, but analysis of exercise dose adherence was not possible because the records of session-by-session intensity compliance were not retained.

## 5 Phase 3: An exploratory, unsupervised machine learning analysis

### 5.1 Cluster analysis

All 17 features measured were normalized using a standard scaler, and the intra-individual differences between baseline and 16-week values were subsequently computed for each feature to generate the corresponding ‘delta_’ features. Subsequently, to mitigate the high feature-to-sample ratio and multicollinearity, principal component analysis (PCA) was performed on the delta features, and the first 8 principal components, which explain 74.55% of the variance, were selected. To analyze exercise-response patterns, clustering was applied to intra-individual change (delta_) features rather than baseline assessments to reduce potential circularity between supervised feature selection and unsupervised clustering. Finally, an exploratory clustering algorithm like K-means, Gaussian Mixture Models (GMM), Hierarchical (Ward/Complete), Spectral, and Density-Based Spatial Clustering of Applications with Noise (DBSCAN) were implemented and optimized through grid search.

### 5.2 Statistical analysis

SPSS version 27 was used to calculate the descriptive statistics of each cluster. Mean ± SD were used for continuous variables, and t-tests were conducted for group comparison. A p-value <0.05 was considered significant. For multiple comparisons, p-values were adjusted using Benjamini–Hochberg False Discovery Rate (FDR), and statistical significance was defined as q < 0.05.

## 6 Results

The Scikit-learn library was used to develop models. Also, Visual Studio Code version 1.98.1 was utilized to conduct the experiments.

### 6.1 Feature selection

**Fig. 2:**
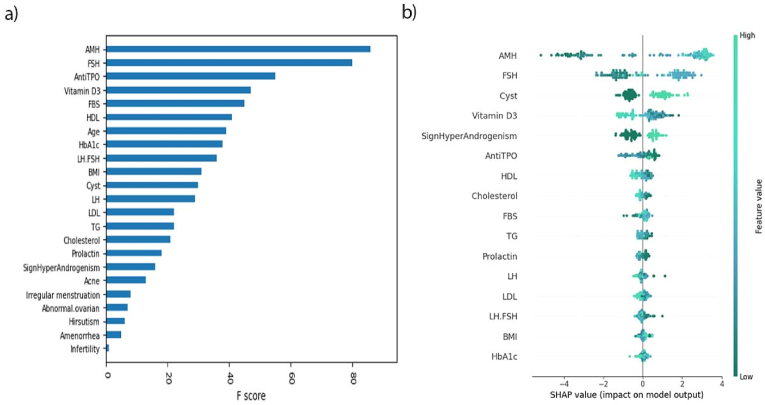
a) XGBoost feature importance; b) Feature impact on model output based on the SHAP value.

As depicted in Fig. 2a, the XGBoost feature importance shows that AMH, FSH, and Anti-TPO have the highest importance, while Infertility, Amenorrhea, and Irregular menstruation have the lowest.

### 6.2 Performance of classifiers with the selected features

This subsection presents the evaluation metrics results of classifiers with the selected features by XGBoost. The results are summarized in Table 1. Overall, following feature selection using XGBoost, the XGBoost model demonstrated the highest predictive performance, with XGBoost achieving 94% accuracy, 97% recall, 90% Specificity, 92% precision, 95% F1-score, and 98% AUC, indicating that the selected features effectively capture the most informative signals for disease detection. Furthermore, SHAP analysis was employed to interpret the contribution of features to the model outcome. As illustrated in Fig. 2b, AMH and FSH showed the strongest positive impact, followed by Cyst, vitamin D3, and hyperandrogenism signs, while metabolic indicators such as lipid profile, HbA1c, and BMI demonstrated moderate effects. These results confirm the central role of reproductive and metabolic factors in the predictive performance of the model, which is along with the selected features by XGBoost.

In the subsequent stage, the model's performance was evaluated by external data. As presented in Table 1, the proposed model has 99% Specificity, 92% Accuracy, 92% Precision, 97% Recall, 95% F1-score, and 91% AUC, which illustrates the optimal performance of XGBoost in comparison to the other trained models.

### 6.3 Results of physical activity intervention

**Fig. 3:**
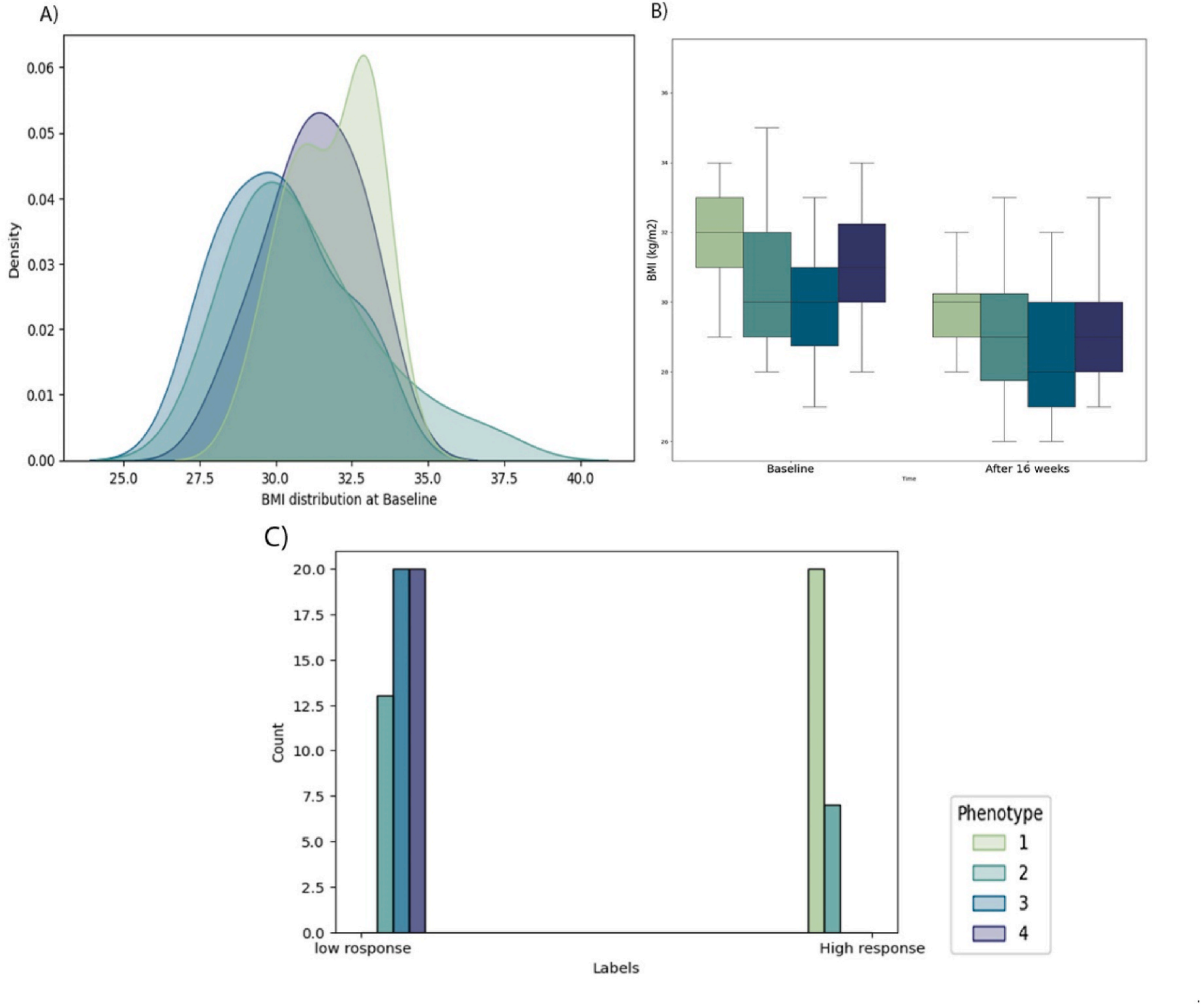
a) Baseline BMI distribution across phenotypes; b) Comparison of BMI changes during intervention; c) Distribution of phenotype counts within each cluster.

Fig. 3a illustrates the baseline distribution of BMI, and Fig. 3b depicts the comparison of BMI changes across phenotypes after 16 weeks of INW. Paired-sample *t*-test illustrated that BMI decreased significantly following 16-week INW (Two-sided p-values = 0.000, t = −12.8) and was effective enough. Supplementary file figure S.I. shows biomarkers at baseline and 16 weeks INW in PCOS patients.

### 6.4 Unsupervised clustering

Table 2presents the comparative validity metrics of the clustering algorithm. As shown, Spectral clustering (gamma = 0.5, k = 2) demonstrated the highest Silhouette score (0.18) and Calinski-Harabasz Index (17.51), suggesting better cluster separation compared to the other models, despite the inherent heterogeneity of the PCOS data. The stability of the Spectral Clustering was assessed via bootstrap resampling (100 iterations), yielding the mean Jaccard of 0.45 (SD = 0.11). However, the stability was moderate; it could suggest the presence of an exploratory and partially reproducible pattern of response in PCOS patients.

**Table 2:** The validity metrics of the clustering algorithm.

Algorithm	Best Params	Silhouette	Davies-Bouldin	Calinski-Harabasz
GMM	K = 2	0.17	2.07	15.80
Hierarchical (Ward/Complete)	K = 2, ward	0.17	1.92	16.50
Spectral	K = 2, gamma = 0.5	0.18	1.93	17.51
K-Means	K = 4	0.15	1.97	12.95
DBSCAN	eps = 2.6, min_s = 2	0.02	3.57	2.95

Spectral Clustering classified the patients into two groups. Gap statistics analysis was performed to determine the optimal number of clusters (Table 3). The results confirmed that the highest Gap value was observed for K = 2 (Gap = 0.327, SE = 0.021), supporting the selection of a two-cluster solution.

**Table 3:** Gap statistics analysis for Spectral Clustering.

K Number of Clusters	Gap Statistics	SE
2	0.327	0.021
3	0.291	0.021
4	0.296	0.022
5	0.295	0.024
6	0.295	0.022

According to the per-cluster silhouette score, Cluster 0 demonstrated a slightly higher mean silhouette score (0.19 ± 0.1) than Cluster 1 (0.17 ± 0.07). As shown in Table 4, the cluster-specific validation metrics demonstrated moderate stability for Cluster 0 and low stability for Cluster 1. According to Table 4, the cluster mean adherence rates (Cluster 0 = 92.26 ± 6.39, Cluster 1 = 93.36 ± 5.84) indicated no differences in exercise compliance and therefore did not influence the observed clustering structure.

**Table 4:** Cluster-specific validation metrics for the selected Spectral Clustering solution.

	size	Mean ± SD Silhouette	Mean ± SD Jaccard	Mean ± SD adherence rate
Cluster 0	53	0.19 ± 0.1	0.48 ± 0.05	92.26 ± 6.39
Cluster 1	27	0.17 ± 0.07	0.28 ± 0.06	93.36 ± 5.84

Taken together, low silhouette values, moderate gap statistic support, and heterogeneous cluster-wise stability illustrated that the observed clusters should be interpreted as weak-to-moderate and exploratory response clusters, and, due to the clustering analysis being exploratory hypothesis generation rather than definitive subgroup identification, both clusters were retained.

Of note, Cluster 1 (n = 27) exhibited the greatest mean improvements (negative changes in features mean reduction) across all biomarkers and was therefore designed as the exploratory high-response pattern. Whereas Cluster 0 (n = 53), showing the lower biomarker improvement, was classified as the exploratory low-response pattern to the exercise. The labels “high-response” and “low-response” were assigned to describe the magnitude of observed biomarker changes following intervention and only represent data-driven cluster designations and should not be considered as clinically validated responder categories.

Table 5 presents a comparison of the mean changes of biomarkers adjusted using the Benjamini-Hochberg FDR correction between clusters. As depicted, significant differences after FDR correction between the two groups were observed in AMH, DHEA-S, FSH, HDL, LH, LH/FSH ratio, total testosterone, and total cholesterol.

**Table 5:** Comparison of mean changes in biomarkers between clusters following 16-week INW.

Features	Mean changes ± SD		t	Two-sided p-values	q-value (FDR)	95% Confidence Interval of the Difference		Cohen Point Estimate
	Cluster 0 (Low-response)	Cluster1 (High-response)				Lower	Upper	
AMH (ng/mL)	0.09 ± 0.45	−0.59 ± 0.58	−5.78	0.000*	0.000*	−1.558	−0.760	−1.368
Anti-TPO (IU/mL)	−0.17 ± 11.35	2.38 ± 9.36	1.00	0.317	0.390	−0.234	0.713	0.238
BMI (kg/m2)	−1.74 ± 1.04	−1.89 ± 1.48	−0.53	0.592	0.592	−0.604	0.347	−0.127
DHEA-S (ng/mL)	−0.28 ± 0.30	−0.60 ± 0.57	−2.70	0.011*	0.022*	−1.282	−0.183	−0.771
FBS (mg/dL)	−11.28 ± 10.29	−16.3 ± 8.89	−2.15	0.034*	0.051	−0.964	−0.038	−0.509
FSH (mIU/mL)	0.04 ± 0.11	0.20 ± 0.10	−6.06	0.000*	0.000*	−1.590	−0.804	−1.434
HDL (mg/dL)	2.72 ± 1.29	4.22 ± 1.40	−4.79	0.000*	0.000*	−1.428	−0.590	−1.134
HBA1c (%)	−0.48 ± 0.81	−0.59 ± 0.45	−0.71	0.478	0.51	−0.542	0.256	−0.141
LDL(mg/dl)	−9.60 ± 6.34	−12.85 ± 6.57	−2.14	0.035*	0.051	−0.962	−0.036	−0.507
LH (mIU/mL)	−0.20 ± 0.15	−0.37 ± 0.22	−3.51	0.001*	0.002*	−1.370	−0.368	−0.941
LH/FSH	−0.10 ± 0.11	−0.30 ± 0.16	−5.82	0.000*	0.000*	−1.698	−0.823	−1.550
Prolactin (mU/L)	−3.74 ± 27.94	−10.62 ± 24.72	−1.08	0.283	0.377	−0.730	0.216	−0.256
TG (mg/dL)	−20.49 ± 15.13	−27.81 ± 11.97	−2.188	0.032*	0.051	−0.971	−0.046	−0.517
Total Testosterone (ng/dL)	−3.64 ± 3.02	−11.67 ± 7.73	−5.19	0.000*	0.000*	−1.773	−0.773	−1.574
Total cholesterol (mg/dl)	−16.89 ± 11.89	−26.89 ± 11.23	−3.62	0.001*	0.002*	−1.244	−0.362	−0.857
Vitamin D3 (ng/Ml)	0.58 ± 7.61	1.87 ± 7.10	−0.72	0.468	0.51	−0.649	0.301	−0.172

*Significant changes between groups (p < 0.05, q < 0.05); q-values were obtained using the Benjamini–Hochberg FDR correction; AMH, Anti-Müllerian Hormone; BMI, Body Mass Index; DHEA-S, Dehydroepiandrosterone sulfate; FBS, Fasting blood sugar; HBA1c, hemoglobin A1C; HDL, high-density lipoprotein; LDL, low-density lipoprotein, TG, Triglycerides; LH, Luteinizing hormone; FSH, Follicle-stimulating hormone, Cohen's d range (small = 0.2, medium = 0.5, large = 0.8).

As shown in Fig. 3c, the high-response exercise cluster was more frequently observed among PCOS phenotypes one and two, and the low-response cluster primarily included PCOS phenotypes three and four. Analysis of biomarker changes following 16-week INW intervention (See supplementary file in Figure S.I.) demonstrated improvements in most metabolic parameters (Figure S.I. (g, h, k-n)) alongside reductions in total testosterone levels (Figure S.I. (i)).

## 7 Discussion

### 7.1 Interpretation of phenotype-specific responses to exercise

In the current study, we analyzed a wide array of integrated non-invasive biomarkers (clinical and non-clinical) and integrated artificial intelligence-based phenotypic clustering approaches before and following the non-pharmacological intervention (the 16-week Nordic interval training) in obese women. According to our results, XGBoost and SHAP feature selection identified AMH, FSH, and Anti-TPO as the most influential predictors. The metabolic indices like HbA1c, BMI, and lipid profiles exhibited moderate effects. Moreover, spectral clustering of the first 8 principal components of intra-individual changes revealed two exploratory response pattern groups: high and low exercise-response. The high-response group was predominantly composed of phenotypes one and two, showing reductions in testosterone and DHEA-s, as well as improvements in HDL and total cholesterol, following the 16-week INW intervention. However, this cluster has relatively low reproducibility (Jaccard = 0.28) and should be interpreted cautiously. Conversely, following the 16-week INW intervention, phenotype four was more frequently observed within the exploratory low-response pattern to exercise. These findings support the hypothesis that responses to non-pharmacological interventions may vary in PCOS phenotypes. This data-driven clustering approach may be useful for exploring heterogeneous response patterns in PCOS patients. However, it should be noted that adherence can influence exercise responsiveness; therefore, the impact of differences in attendance or compliance on the observed cluster structure cannot be excluded.

Our clustering solution, with a mean Jaccard index of 0.45, demonstrated moderate stability, suggesting that the observed response patterns should be considered as exploratory and hypothesis-generating rather than definitive biological groups. Also based on cluster-specific validation (Table 4), Cluster 0 (Jaccard = 0.48) indicated higher stability than Cluster 1 (Jaccard = 0.28). The results suggest that the high-response group may be less reproducible and should be cautiously interpreted.

### 7.2 Clinical implications of androgen reduction

From a clinical perspective, participants exhibited a reduction in androgen excess and improvements in metabolic outcomes following the 16-week INW intervention. As reflected in Table 5 and Fig. 3c, the higher mean differences in total testosterone and DHEA-s are related to the high-response cluster, which is predominantly composed of phenotypes one and two. Reductions in testosterone and DHEA-s highlight the beneficial impact of exercise on signs of hyperandrogenism as a core feature of PCOS, especially in phenotypes one, two, and three, where hyperandrogenism is a criterion. Many studies have indicated the impact of exercise on the reduction of testosterone and DHEA-s in PCOS women, regardless of phenotypes.^18^ The present study supports the hypothesis that exercise responsiveness may vary across PCOS phenotypes. Thereby, supporting the use of ML-based clustering may be useful for generating hypotheses regarding individualized intervention design. However, further validation is needed to confirm these subgroup-specific effects.

### 7.3 Metabolic benefits and phenotype four response

Regular exercise, particularly INW, may be recommended as a first-line intervention for hyperandrogenic phenotypes, notably phenotype one. Improvements in BMI, HbA1c, and lipid parameters demonstrate the potential of lifestyle-based strategies to mitigate metabolic risk factors associated with PCOS. These findings are in agreement with evidence that predominantly shows exercise can improve insulin sensitivity and reduce obesity,^21^ and metabolic consequences are more prevalent in the hyperandrogenic PCOS phenotype (phenotypes one, two, and three) compared to the normoandrogenic phenotype (phenotype four).^2^ Phenotype four, characterized by oligo/anovulation and polycystic ovarian morphology without hyperandrogenism, was more frequently represented within the exploratory low-response pattern. Consistent with our results, a systematic review reported limited improvement in ovarian morphology despite consistent aerobic training programs.^22^ The relatively modest response in phenotype four may suggest that mechanisms underlying this subtype could differ from hyperandrogenic phenotypes, which may be less driven by metabolic dysregulation and more by intrinsic ovarian factors; however, further validation is required. While the observations may suggest biological differences between PCOS phenotypes, because of the methodological framework used for biomarker selection, the interpretation of the clustering results should be cautious.

These findings are consistent with previous evidence highlighting the central role of gonadotropins and ovarian biomarkers in PCOS pathophysiology.^23^ The biomarkers selected were chosen based on their PCOS classification ability in phase 1. Although clustering was performed on longitudinal changes (delta values) and no labels or supervised learning output from Phase 1 were utilized in the clustering process, the dependence between Phase 1 and Phase 3 cannot be completely excluded.

The suggested clusters should be interpreted as exploratory response patterns that demonstrate a combination of underlying heterogeneity of the PCOS phenotype and responses to the exercise.

However, further studies are needed to evaluate clustering using biomarker sets optimized for exercise intervention and other feature selection strategies.

### 7.4 Role of explainable AI in biomarker selection

Furthermore, SHAP analysis confirmed the robustness of feature selection by demonstrating that AMH and FSH exerted the strongest positive contributions to the model, followed by Anti-TPO and vitamin D3. In contrast, metabolic markers such as HbA1c, BMI, and lipid profiles had moderate but clinically relevant effects. Taken together, the explainability of our models underscores the importance of integrating both reproductive and metabolic domains for reliable PCOS management and the benefits of exercise for PCOS management.

## 8 Limitation and future directions

Several strengths and limitations of the current study warrant discussion. A key strength of this study lies in the integration of real-world, non-invasive clinical and non-clinical biomarkers with an advanced artificial intelligence-based analysis to explore the pattern of phenotypic responses to INW in obese women with PCOS. This approach could suggest the identification of potentially distinct response patterns while maintaining clinical applicability. Nevertheless, the identified response clusters should be interpreted with caution. Biomarkers were selected based on their discriminative performance in PCOS classification, and clustering was performed on exercise intervention-induced biomarkers rather than baseline measurements. Consequently, it remains possible that part of the observed cluster reflects underlying PCOS-related biological heterogeneity in addition to exercise-induced adaptations. Therefore, the observed cluster should be interpreted as exploratory exercise-response profiles rather than a definitive biological phenotype.

However, some methodological limitations should be acknowledged. First, the intervention phase was a prospective single-arm design, and the absence of a parallel control group is an important limitation. Although significant biomarker changes were observed following the 16-week INW intervention, causal attribution to the intervention cannot be established.

Although the biomarker changes were observed following the 16-week INW, these changes cannot be attributed to the intervention itself. Unmeasured confounding factors like regression to the mean, behavioral changes, seasonal influences, and temporal variation cannot be excluded. Accordingly, the findings should be interpreted as associations observed in the 16-week INW intervention rather than definitive evidence of intervention effectiveness. To establish causal relationships between INW and biomarker changes across PCOS phenotypes, future randomized controlled trial studies with an appropriate control group are required.

Second, these results were obtained in a population of obese individuals in the hospital and cannot automatically be generalized to another population. Whereas the integrated artificial intelligence-based phenotypic clustering approaches used may be transferable to broader clinical and demographic populations.

Third, some characteristics that may influence PCOS phenotypes, such as genetics and other biological factors, have not been considered.

Fourth, the clustering solution represented only moderate stability (mean Jaccard index of 0.45), suggesting that exploratory response patterns may exist, but the boundaries between phenotypes may be fluid rather than rigid.

Finally, exercise intensity was monitored throughout the 16-week INW using direct supervision and heart rate monitoring; records of session-by-session compliance with the prescribed intensity range were not available. Therefore, we could not quantify the effects of dose-response on exercise compliance, and consequently, the potential influence of exercise adherence on intervention outcome could not be evaluated.

Overall, these findings are considered exploratory. Future studies with larger, multi-ethnic cohorts and longer follow-up durations are warranted to validate these results and to elucidate the mechanistic pathways linking exercise, hormonal regulation, and metabolic improvement in PCOS.

## 9 Conclusion

Our machine learning-based clustering suggested two exploratory response patterns following 16 weeks of INW. The exploratory high-response pattern was more frequently observed in hyperandrogenic PCOS phenotypes (one, two) and was associated with larger improvements in androgenic and metabolic biomarkers. Conversely, phenotype four was more commonly found on the exploratory low-response pattern. These findings can suggest potential heterogeneity in PCOS phenotypes' response to exercise. From an interventional perspective, a 16-week interval Nordic walking (INW) program was associated with reductions in hyperandrogenic and metabolic biomarkers, although, due to the absence of a control group, causal attribution cannot be established. These findings generate the hypothesis that phenotype-specific exercise responses may exist and also support further evaluation of INW as a first-line lifestyle intervention for hyperandrogenic PCOS. However, given the exploratory nature of the clustering results and modest cluster validity metrics (Silhouette = 0.18; Jaccard = 0.45), these findings should be interpreted as hypothesis-generating and a foundation for personalized, non-pharmacological PCOS management strategies and should not be interpreted as definitive biological phenotypes. Further validation in larger, independent cohorts is needed.

## Data statement

Due to the sensitive nature of the clinical data used to train and evaluate the AI models, the datasets cannot be shared publicly. However, methodological details and model descriptions are provided to ensure reproducibility.

## Ethics approval

This study was performed in line with the principles of the Declaration of Helsinki. Approval was granted by the University of Mazandaran Institutional Ethics Committee (IR.UMZ.REC.1402.037)

## Author contribution

Study concept and design: V.DR; acquisition of data: M.M and Z.AT; analysis and interpretation of data: M.M and Z.AT; drafting of the manuscript: V.DR and Z.AT; critical revision of the manuscript: V.DR; statistical analysis: Z.AT; and study supervision: V.DR. All authors read and approved the final manuscript.

## Declaration of generative AI and AI-assisted technologies in the manuscript preparation process

None.

## Funding

This work is based upon research funded by the 10.13039/501100003968Iran National Science Foundation(INSF) under project No. 4037411.

## Declarations of interest

None.
