# Supplementary material for: Clustering of non-invasive biomarkers to distinguish phenotype-related response differences in women with PCOS after the 16-week Nordic interval training intervention: A prospective cohort study
Source: J Exerc Sci Fit. 2026 Jul 17;24(4):200499. doi: 10.1016/j.jesf.2026.200499 (PMC13452378; doi:10.1016/j.jesf.2026.200499)
Supplement: Supplementary file 1 [file mmc1.docx]

**Table S.I.** Feature analysis of original data instances and feature distribution

| No. | Feature | Description | Mean | Std |
| --- | --- | --- | --- | --- |
| 1 | **PCOS (Y/N)** | Class label (yes PCOS or no PCOS) | NA | NA |
| 2 | Age (yrs) | Patient’s age in years | 35.44 | 6.36 |
| 3 | Weight (kg) | Patient’s weight in Kg | 69.55 | 12.36 |
| 4 | Height (cm) | Patient’s Height in Cm | 158.93 | 5.92 |
| 5 | BMI (kg/m^2^) | Body mass index of a patient (height/weight) | 27.53 | 4.73 |
| 6 | Abnormal ovaries (Y/N) | Ovarian abnormality status (yes or no) | NA | NA |
| 7 | Ovarian cysts (Y/N) | Existence of cysts in the ovary (yes or no) | NA | NA |
| 8 | Dysmenorrhea (Y/N) | Existence of primary or secondary dysmenorrhea (yes or no) | NA | NA |
| 9 | Amenorrhea (Y/N) | Existence of primary or secondary amenorrhea (yes or no) | NA | NA |
| 10 | Infertility (Y/N) | Failure to achieve a pregnancy after 12 months | NA | NA |
| 11 | Irregular menstruation (Y/N) | Irregularity of menstrual cycle (yes or no) | NA | NA |
| 12 | SignHyperAndrogenism (Y/N) | Existence of signs of hyperandrogenism (yes or no) | NA | NA |
| 13 | Acne (Y/N) | Existence of acne (yes or no) | NA | NA |
| 14 | Hirsutism (Y/N) | Extra hair growth caused by excess androgen (yes or no) | NA | NA |
| 15 | AMH (ng/mL) | Anti-Müllerian hormone | 2.85 | 3.14 |
| 16 | FSH (mIU/mL) | The follicle-stimulating hormone | 15.18 | 23.67 |
| 17 | LH (mIU/mL) | Luteinizing hormone | 11.44 | 17.45 |
| 18 | Prolactin (ng/mL) | Prolactin hormone | 23.81 | 55.08 |
| 19 | TSH )mIU/L( | The thyroid-stimulating hormone | 2.74 | 7.39 |
| 20 | Anti-TPO (IU/ml) | Antithyroid Peroxidase Antibody | 60.27 | 170.71 |
| 21 | HbA1c (%) | Hemoglobin A1C | 5.45 | 0.67 |
| 22 | FBS (mg/dL) | Fasting blood sugar | 95.52 | 12.51 |
| 23 | TG (mg/dL) | Triglyceride | 128.38 | 53.38 |
| 24 | Cholesterol (mg/dL) | Total [Cholesterol](https://resalathospitallab.com/interpretation-of-tests/general-health/chol-tg-hdl-ldl/%D8%AA%D9%81%D8%B3%DB%8C%D8%B1-%D8%A2%D8%B2%D9%85%D8%A7%DB%8C%D8%B4-cholesterol.html) | 181.88 | 31.64 |
| 25 | LDL (mg/dL) | Low-density lipoprotein | 106.71 | 30.73 |
| 26 | HDL (mg/dL) | High-density lipoprotein | 47.91 | 12.63 |
| 27 | VITD3 (ng/mL) | Serum Vitamin D3 Level | 27.48 | 13.61 |
| 28 | AFC_right/left_ | The number of antral follicles (resting follicles) in the ovaries | 12 | 3.5 |
| 29 | Progesterone (ng/mL) | Progesterone hormone | 1.06 | 5.35 |
| 30 | B-HCG (mIU/ml) | B-HCG hormone | 0.62 | 1.26 |
| 31 | Abortion (Y/N) | Pregnancy doesn’t result in the birth of a child (yes or no) | NA | NA |

Figure S.I. Biomarkers distribution at baseline and after 16 weeks INW
